# Long-Term Soft Denture Lining Materials

**DOI:** 10.3390/ma7085816

**Published:** 2014-08-12

**Authors:** Grzegorz Chladek, Jarosław Żmudzki, Jacek Kasperski

**Affiliations:** 1 Division of Materials Processing Technology, Institute of Engineering Materials and Biomaterials, Silesian University of Technology, ul. Konarskiego 18a, Gliwice 44-100, Poland; E-Mail: jaroslaw.zmudzki@polsl.pl; 2 Department of Prosthetic Dentistry, Medical University of Silesia, pl. Akademicki 17, Bytom 41-902, Poland; E-Mail: kroczek91@interia.pl

**Keywords:** dental materials, dentures, polymers, edentulism, soft lining, *Candida albicans*

## Abstract

Long-term soft denture lining (LTSDL) materials are used to alleviate the trauma associated with wearing complete dentures. Despite their established clinical efficacy, the use of LTSDLs has been limited due to the unfavorable effects of the oral environment on some of their mechanical and performance characteristics. The unresolved issue of LTSDL colonization by *Candida albicans* is particularly problematic. Silicone-based LTSDL (SLTSDL) materials, which are characterized by more stable hardness, sorption and solubility than acrylic-based LTSDLs (ALTSDLs), are currently the most commonly used LTSDLs. However, SLTSDLs are more prone to debonding from the denture base. Moreover, due to their limitations, the available methods for determining bond strength do not fully reflect the actual stability of these materials under clinical conditions. SLTSDL materials exhibit favorable viscoelastic properties compared with ALTSDLs. Furthermore, all of the lining materials exhibit an aging solution-specific tendency toward discoloration, and the available cleansers are not fully effective and can alter the mechanical properties of LTSDLs. Future studies are needed to improve the microbiological resistance of LTSDLs, as well as some of their performance characteristics.

## 1. Introduction

Long-term soft denture lining (LTSDL) materials constitute a group of polymer materials that can remain in the oral cavity for at least four weeks; in practice, however, their use can extend to several months or even years. The use of LTSDLs is mostly recommended in edentulous patients with sharp or atrophied alveolar ridges, in patients with thin atrophic mucosa, in patients in whom the mucosa presents insufficient tolerance to the load transmitted by the dentures or who experience pain at nerve ending locations, in cases of the formation of recurrent sore spots under the dentures, in cases in which the denture exhibits poor retention, as well as for relining in implantology and to perform postoperative obturation [1,2,3,4]. The application of a soft material is intended to increase the comfort of denture wearers and to support prosthetic treatment. LTSDLs can help to evenly distribute the biting loads transferred onto the soft tissues during chewing and to relieve the mucosa from high mechanical stress [5]. Note that LTSDLs cannot, as has sometimes been suggested, reduce the forces transmitted by the denture-bearing area, as clearly explained by Braden* et al.* [5].

Commercially available dental materials include silicone-based long-term soft denture linings (SLTSDLs), acrylic-based long-term soft denture linings (ALTSDLs) and, sporadically, materials based on other polymers. Currently, polymethacrylate materials are used less frequently and are available as two-component powder-liquid systems. SLTSDLs are available as one-component materials that cross-link at high temperatures and as two-paste A-type silicone systems that cross-link at room temperature [6]. Light-cured urethane acrylate and a polyphosphazene elastomer compounded with methacrylate monomers are other examples of materials used as LTSDLs [7].

In this paper, we review the basic mechanical and functional properties of LTSDLs as determined from their chemical compositions, with particular emphasis on implementing laboratory findings in clinical practice and possible future improvements of their characteristics.

## 2. Compositions of LTSDLs

Silicones used as soft lining materials are classified as autopolymerized (RTV—room temperature vulcanized) or heat-cured (HTV). Examples of autopolymerized SLTSDL materials include Permaflex Ufi Gel SC, Ufi Gel P, Mollosil Plus, Mollosil, Tokuyama Soft, Mucopren soft, Dentusil, GC Reline Soft, GC Reline Ultrasoft, and Sofreliner Tough Mucosoft. These materials are two-paste systems. A typical “catalyst paste” is a mixture of vinyl-terminated polydimethylsiloxanes with a platinum catalyst. The “base paste” consists of vinyl-terminated polydimethylsiloxanes with hydride-terminated polydimethylsiloxanes. The hydrosilylation reaction begins after both components are mixed, and the addition of Si–H from the hydride functional siloxanes results in bonds across the unsaturated bonds, forming vinyl functional siloxanes [8]. This curing reaction does not produce by-products. The reaction of vinyl functional siloxanes with hydride functional siloxanes occurs at a 1:1 stoichiometry; however, note that when functionalized fillers are used, the ratio of hydride to vinyl is much higher. The optimal cure ratio can be determined, for example, by measuring the hardness of cured samples obtained using different ratios. Due to the low temperature required for cross-linking, these SLTSDLs can be cured in the mouth and are thus called “chair-side” SLTSDLs. However, the final hardness of these materials is often obtained after a few hours and is generally lower than that of HTV silicone [9]. The use of HTV silicone in soft linings, such as in Molloplast B, Permaflex, Flexor, and Luci-Soft, has frequently been investigated. The typical materials, which cross-link with radicals, consist of polydimethylsiloxanes and an organic peroxide, such as benzoyl peroxide. Cross-linking is initiated by free radicals that are generated by the decomposition of organic peroxides at high temperatures. Effective cross-linking can only be achieved when some vinyl groups are present on the polymer chains [8]. The mechanical properties of silicone materials without fillers are generally insufficient for most applications; thus, fillers such as nanoscale amorphous fumed silica are commonly used. The application of properly surface-treated silica fillers, with concentrations of up to few dozen percent, results in the formation of bonds between the filler and the silicone polymer and increases the hardness and strength [10]. In some studies, silica fillers have been experimentally incorporated into LTSDLs to enhance their mechanical properties. However, at the laboratory level, it is difficult to obtain a homogenous dispersion of filler without aggregations [10], which can reduce the expected effects of the filler addition. Commercially used mixing machines use vacuum and high shear action to improve the incorporation of fillers into high-viscosity materials and to allow trapped air and large aggregations to be eliminated [8]. Note that the compounding of the filler to the polymer also increases the viscosity.

Acrylates are available as two-component powder-liquid systems. The “powder” is typically poly(ethyl methacrylate) with a radical initiator (*i.e.*, organic peroxide). The composition of the “liquid” component for acrylic-based denture lining materials depends on the intended application [11,12]. The “liquid” component for ALTSDLs contain a high-molecular-weight monomer, such as ethyl methacrylate, *n*-propyl methacrylate or *n*-butyl methacrylate; a plasticizer, such as aromatic esters (e.g., dibutyl phthalate) or ethyl acetate; and a dimethacrylate cross-linking agent, such as ethylene glycol dimethacrylate [11,13]. Plasticizer molecules can separate polymer chains, which lowers the glass transition temperature and, thus, makes the material softer [12]. However, such plasticizers are not “bound” within the resin; therefore, they leach out of the resin, which is one of the primary reasons for changes in the mechanical properties of ALTSDLs [11]. The use of higher molecular weight acrylic monomers in ALTSDLs allows softer materials to be produced because after polymerization, the resulting materials possess a lower glass transition temperature. Consequently, less plasticizer is required, and the detrimental effects of plasticizer leaching can be reduced [11,14]. Parker* et al.* [15,16] also reported that polymerizable plasticizers such as dioctyl maleate cannot leach out of the resin as easily as conventional plasticizers and that soft lining materials containing such plasticizers possess appropriate mechanical properties. Light-cured urethane acrylate (Triad Resiline) and a polyphosphazene elastomer compounded with methacrylate monomers (Novus) are additional examples of materials used as LTSDLs [7].

The chemicals that are released from LTSDLs are dependent on the chemical formulations of the base materials or on the amounts of additives that were leached; therefore, the composition of the chemicals that are released is highly differentiated for various materials [13,17]. Over the past decade, there have been few studies on this issue. Brożek* et al.* [13,17] divided the compounds released from soft liners stored in denture cleansers and artificial saliva into three categories: (1) monomers, such as methyl methacrylate (MMA), ethyl methacrylate (EMA), ethylene glycol dimethacrylate (EGDMA), and dodecyl methacrylate; (2) additives, such as diethyl phthalate (DEP), dibutyl phthalate (DBP), and tributyl acetylcitrate (TBC); and (3) compounds formed in the reactions of the ingredients used in soft liners, such as benzene, toluene, benzophenone, 2,6-bis(1,1-dimethylethyl)-4-(1-oxopropyl) phenol, diphenyl ether, and isovaleric anhydride. In these studies, more chemicals leached from the examined ALTSDLs than from the examined SLTSDL. As expected, monomers and many additives (plasticizers) were identified in the ALTSDLs. Additionally, products from the decomposition of benzoyl peroxide were detected, such as benzene, toluene, diphenyl ether and benzophenone. In Villacryl Soft, 2,6-bis(1,1-dimethylethyl)-4-(1-oxopropyl) phenol was produced as a product from the oxidation of stabilizer [17]. In the SLTSDL materials examined in those studies, isovaleric anhydride, dodecyl methacrylate, diphenyl ether, benzene, toluene and dibutyl phthalate were present. In silicone Molloplast B, benzophenone, a product from the decomposition of benzoyl peroxide, was also detected [13]. EGDMA was identified in both SLTSDLs and ALTSDLs by Brożek* et al.* [17]; however, EGDMA was not identified in SLTSDLs in their previous study [13]. The authors concluded that the presence of EGDMA, which was added to improve cross-linking, could suggest that the cross-linking process was incomplete or that the cross-links were not stable [13]. Sofou* et al.* [18] investigated the release of residual monomers from LTSDLs with the use of HPLC (high-performance liquid chromatography). Methyl methacrylate was not detected in the examined SLTSDLs; however, methacrylic acid and different phthalates were detected in all of the materials. The SLTSDLs generally exhibited lower monomer concentrations than the ALTSDLs. Quantitative and qualitative differentiation of the chemicals leaching into the environment indicates that their release should be more extensively investigated, especially considering that some of these chemicals may be toxic [19].

## 3. Clinical Effects of Long-Term Soft Denture Linings

**Figure 1:**
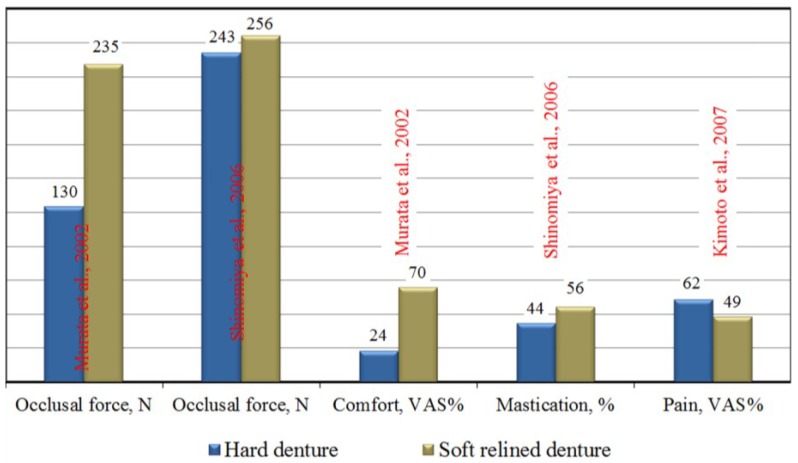
The effects of soft denture linings on indicators of functional efficiency, in which the VAS is a visual analog scale of the patients’ subjective assessments of satisfaction, based on [22,25,27,28].

Although LTSDLs have rarely been subjected to clinical trials, there are a few studies on their use. As many as 93% of patients participating in a six-year clinical study conducted by Schmidt* et al.* [20,21] reported that dentures with soft linings were more comfortable than hard acrylic dentures. The use of LTSDLs is characterized by markedly improved speech and ability to chew, significantly reduced feelings of pain and oral soreness under the dentures, better retention and stability of the dentures, an increase in psychological comfort and longer denture wearing times [22,23,24]. The maximum occlusal force can significantly increase with LTSDLs [25,26]; however, other investigations have not confirmed this finding [27,28]. An example showing the effects of using LTSDLs on indicators of functional efficiency is presented in Figure 1. Analogous benefits were reported when LTSDL materials were used to retain an existing denture with implants [29], in which the use of a soft material to retain the denture resulted in a relatively large amount of work required to separate the attachment, despite the low values of the retention forces [30].

**Figure 2:**
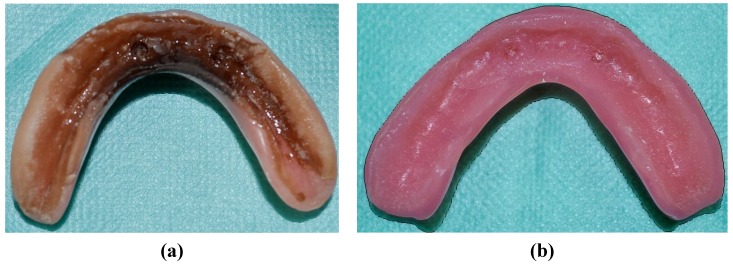
Soft-lined dentures (Molloplast B) after 19 months of use by a smoking and coffee-drinking patient (**a**) and after 10 months of use by a patient maintaining good oral hygiene (**b**). Strong discoloration (**a**) and numerous microbial colonies (**b**) can be observed.

Although the longevity of SLTSDLs was confirmed under clinical conditions, the presence of discoloration and odor was frequently reported among tobacco smokers [7,8,9,10,11,12,13,14,15,16,17,18,19,20,21,31]. Moreover, a gradual increase in the number of fungal colonies on the surfaces of the linings was observed during the first year of use, along with slight discoloration in non-smokers and the local debondings of LTSDLs from the denture base [32]. Examples of soft denture linings with discoloration and dentures colonized by microorganisms are presented in Figure 2.

The aforementioned clinical trial results should be considered particularly important in light of the increase in the number of edentulous patients expected in future years [33]. Due to economic and sometimes also medical and psychological reasons, a considerable number of these patients will not be subjected to expensive [22] and error-prone treatment with dental implants. These patients will continue to use acrylic dentures that can cause pain and mucosal abrasions [22]. The use of LTSDLs will facilitate their adaptation to new dentures [23] and improve the process of rehabilitation, which should be reflected by fewer potential denture corrections and a resulting decrease in treatment costs. Thus, LTSDLs constitute a prospective group of dental materials, and previously reported clinical problems with LTSDLs [7,8,9,10,11,12,13,14,15,16,17,18,19,20,21,31,32] justify further research aimed at improving their properties.

## 4. Colonization of Soft Denture Lining Materials by Microorganisms

The conditions under the denture base promote the growth of microorganisms. High humidity and temperature, as well as inaccessibility for self-cleaning by saliva, promote the replication of bacteria and fungi. The properties related to the colonization of LTSDLs by *C. albicans* have been widely investigated for SLTSDLs; however, the colonization of ALTSDLs has been less well researched. There have been many studies on temporary acrylic soft linings.

Mutluay* et al.* [32] demonstrated that after a half year of use under clinical conditions, fungal colonies were present on one-third of the examined silicone linings on average. However, no failures due to fungal colonization were noted. After 12 months, fungal colonies were detected on as many as 44% of the linings on average, and a total of 31% of the recorded failures were due to fungal colonization. However, the intensity of colonization varied markedly depending on the type of material used. These findings were consistent with the results of* in vitro* studies, which showed that none of the available LTSDL materials were resistant to colonization by pathogenic fungi [1]. Although* in vitro* inhibition of fungal growth was documented in a few studies of Molloplas-B material [34,35], other authors did not confirm such an effect; thus, the aforementioned antifungal effects likely resulted from the release of dibenzoyl peroxide into the environment [1,36].

Saliva and salivary proteins have been identified as factors that promote the colonization of soft linings by *C. albicans* [34,37,38,39]. The fungi initially adhere to the LTSDL surface [40,41]. Bulard* et al.* [1] conducted a comprehensive study on the colonization and penetration of LTSDLs by *C. albicans*. These researchers observed that after one hour of incubation in a standardized cell suspension, there was no significant difference between the numbers of *C. albicans* cells adhering to the smooth surfaces of the examined ALTSDL and SLTSDL materials. During the penetration test, the LTSDL samples that were bonded to the denture base resin were incubated for six weeks in artificial saliva with *C. albicans* cultures. Subsequently, cross-sections of the samples, including their external layers (surface), central sections and layers adjacent to lining-denture interface, were investigated under an epifluorescence microscope. Both the surface and central section were observed to have been colonized. In an investigation of the heat curing of SLTSDLs, the penetration of *C. albicans* was relatively comparable, which was explained by the similar chemical compositions of the linings; however, for ALTSDLs (EverSoft), a considerably lower level of penetration by blastospores and hyphal forms was noted. The authors related this effect to the presence of free ethyl alcohol within the cured ALTSDL samples and to its inhibitory effects on yeast growth during long-term exposure. Additionally, a high degree of colonization was also observed adjacent to the denture base material-SLTSDL interface, suggesting that microbiological contamination could have been a potential cause for the debonding of SLTSDLs from dentures in the oral cavity. These findings suggested that the efficacy of cleaning with widely available chemical agents could be limited for soft linings because the contaminated central section of the material still constituted a reservoir of microorganisms. Moreover, the number of blastospores that penetrated through the LTSDLs was smaller than that for a hard acrylic liner, and the latter was not penetrated by fungal hyphae [1]. These observations were consistent with the results of other studies [42,43] and suggested that the soft structure of LTSDLs could promote colonization.

Nevzatoğlu [42] reported that HTV SLTSDLs exhibited significantly less yeast adhesion than RTV linings and related this effect to the greater hydrophilicity of the latter linings. Kang* et al.* [44] reported that ALTSDL materials exhibited greater *Candida* adhesion than SLTSDLs and attributed this result to the base components and the degree of hydrophilicity of the materials. The relationship between the surface roughness of LTSDLs and the adhesion of *C. albicans* to these materials has raised some controversies. Some authors [1,40,45] have demonstrated that samples with smooth surfaces are less susceptible to fungal colonization, and Taylor* et al.* [46] suggested that an increase in LTSDL roughness during use could increase the susceptibility of these materials to microbial colonization. Nevertheless, other authors did not confirm the association between the roughness of the surfaces of the linings and the degree of their fungal colonization [35,36]. Thus, roughness may not be the sole determinant of colonization, and its importance in this process remains unclear. In addition, the association between the surface free energy of LTSDL materials and the adherence of *C. albicans* is not straightforward. Although no correlation between the level of surface free energy and adherence of most *C. albicans* strains has been documented, one strain has been shown to have a significantly higher level of adherence to materials with lower surface free energies [41]. Therefore, Waters* et al.* [41] suggested that the degree of *C. albicans* adherence was determined by the characteristics of the fungal cell surface.

A large number of thermal cycles could promote the growth and colonization of yeast [34,39]. Boscato* et al.* [47] found that the use of inappropriate cleaning agents promoted the formation of grooves and fissures on material surfaces; in addition, the removal of adhered microorganisms from these structures was difficult. In addition, Huh* et al.* [48] found that SLTSDLs stored in some denture cleaners exhibited a greater ability for forming yeast biofilms than specimens stored in distilled water. Faccio* et al.* [49] observed that the level of biofilm formation in elderly patients with controlled diabetes was identical to that in healthy persons.

The aforementioned evidence suggests that microbial colonization of LTSDLs constitutes a principal and still unresolved issue associated with their exploitation. Thus far, studies have not unambiguously determined the properties of LTSDL materials that determine their susceptibility to fungal colonization, which has hindered the identification of a material that would be optimal in this context. The incorporation of nystatin into lining materials can be effective, but only for a short period [50]. In light of the previously mentioned findings of Bulard* et al.* [1], a study aimed at designing composite LTSDLs that are resistant to microbial penetration appears highly justified. The studies addressing the problem in question revealed the increased antimicrobial resistance of SLTSDL materials with nanosilver [51,52], with the concentrations of the silver nanoparticles ranging between 20 ppm and 200 ppm. However, the longevity of the achieved effect has not yet been analyzed. However, for other dental materials, Sokołowski* et al.* [53] demonstrated that the number of silver ions released into the environment decreased over time, which could reduce the antimicrobial effects. Moreover, the incorporation of nanosilver was shown to affect the mechanical and performance properties of the analyzed composites, while also causing discoloration [52]. Because color represents an important property of LTSDLs, the use of white ceramic micro- or nanofillers with antimicrobial properties appears to be a more appropriate solution to the problem in question.

## 5. The Hardness of LTSDLs

Stability of hardness during use is a desirable feature of LTSDL materials because any increase in hardness can worsen the distribution of the masticatory load and lower the absorption of elastic energy, which is transmitted onto the mucosal membrane under dentures, thereby exacerbating the clinical problems experienced by patients [24,54]. The hardness of LTSDLs is determined using the Shore A method, and standardized procedures for testing have been defined by the respective ISO [55] and ASTM (American Society for Testing and Materials) standards [56]. However, only the ISO standard is directly dedicated to LTSDL materials and includes rules for classifying these materials as soft or extra-soft. The Shore A hardness values after 24 h of aging in distilled water at 37 °C for soft and extra-soft materials should range from 25 to 50 units and be less than 25, respectively; after 28 days of aging, the Shore A hardness values should be less than 55 and not greater than 35, respectively. Siddiqui* et al.* [57] reported that investigations for different aging protocols should be performed with samples that are at least 6 mm thick (required by the ISO and ASTM standards). Thinner samples will yield incorrectly high values, and the error will increase as the thickness decreases. The data obtained for thinner samples require further attention because these measurements, although interesting for comparative purposes within particular studies, did not result in Shore A hardness measurements in accordance with the ASTM and ISO standards. The initial hardness values of different LTSDLs measured with a Shore A durometer are listed in Table 1. This list contains results from the works cited in this review. However, even for the same materials with samples of the correct thickness, the hardness values were different in some cases. This finding could be associated with the period between the end of the curing procedure and initial measurements or with potential changes in the chemical compositions by the manufacturers over time. Therefore, only the effects of various factors on the changes in hardness are described in this review, but usually without their values.

Laboratory studies have documented increases in ALTSDL hardness by as much as 150% during the initial six months of aging in distilled water [58,59,60,61]. In contrast, the hardness of SLTSDLs that were aged in distilled water was altered by no more than 63%, or it remained unchanged [52,58,59,60,61,62]. Polyzois* et al.* [61] investigated the hardness changes of two ALTSDLs over one year of aging in distilled water: one heat-cured (Super-Soft) and one autopolymerized (EverSoft). The heat-cured material was considerably harder than the autopolymerized material. However, the hardness of Super-Soft was 78.4; thus, in accordance with the ISO, this material should not be classified as a soft lining material. The change in hardness for the heat-cured material after the first month was approximately 8%, after which the value was stable. However, for the autopolymerized EverSoft, the changes were approximately 120% after the first month and approximately 150% after five months, at which point a plateau was reached. The changes in hardness were caused by the leaching of plasticizers. Parr* et al.* [62] showed that the hardness of the autopolymerized SLTSDL material was lower than that of laboratory-processed material and remained generally constant throughout the storage period because only hardening during the first 24 h was observed, which could have been caused by a more complete polymerization reaction. The hardness of the heat-cured SLTSDL increased gradually for a half-year by approximately 8%. Amnuay* et al.* [9] reported that some HTV silicone-based materials could soften during one year of aging in distilled water, most likely attributed to the washing out of inner fillers; however, this observation requires further verification. Kim* et al.* [63] investigated Shore A hardness in accordance with the ISO standards for an autopolymerized ALTSDL and for six autopolymerized SLTSDL materials. During the 28 days of aging in distilled water at 37 °C, the hardness increased for the ALTSDL Durabase by 34%; however, for the SLTSDLs, the hardness increased from 5% (Mucosoft) to 63% (GC Reline Ultrasoft). The authors attributed the hardness changes to the gradual leaching of soluble contents from the materials. Similar hardness changes for GC Reline Ultrasoft were reported by Iwaki* et al.* [64]. The considerable differences in the profiles of the hardness changes for silicone-based and acrylic materials could result from their chemical compositions. The initial low hardness values of ALTSDLs are achieved due to the use of plasticizers that are further leached during aging. In contrast, the desired hardness of SLTSDLs is modulated by the degree of their cross-linking or by the addition of fillers. However, the results for SLTSDLs show significant differences that could be caused by the material composition, chemistry or polymerization procedure, and without access to proprietary knowledge, it is difficult to assess the effects of the material composition on the hardness changes reported in the presented works.

Santawisuk* et al.* [10] investigated addition-cured, medical-grade RTV silicone with 15%–40% trimethylated silica after the incorporation of 2% to 10% of the hydrophobic filler AEROSIL^®^ R 812S (Degussa, Cheshire, UK); the results revealed a significant and gradual increase in Shore A hardness and in the other mechanical properties mentioned below; however, the viscosity was acceptable to a concentration of 6% of the new filler.

Pavan* et al.* [65] observed that a small amount of disinfection with microwave energy and different chemical solutions slightly altered the hardness values of SLTSDLs but exerted no effects on the ALTSDL EverSoft. Nevertheless, more disinfection cycles could lead to hardening of the materials [66].

The use of 2000 thermal cycles with sudden changes in temperature in the range from 5 to 55 °C increased the SLTSDL hardness from 4% to 15%, most likely associated with further cross-linking occurring during the experiment [67]. This hypothesis should be verified by analyzing the relationship between the number of thermal cycles and the material hardness.

**Table 1:** The initial hardness values of the long-term soft denture linings (LTSDLs), measured for samples with different thicknesses at the beginning of different experiments.

Source	Material/type	Samples thickness, mm	Hardness, Shore A units
Yoeli, Z.* et al.* [58]	Molloplast B/heat-cured silicone	8	46
	Permaflex/heat-cured silicone		45
	Permasoft/autopolymerized acrylic		28
	Flexacryl/autopolymerized acrylic		56
Canay, S.* et al.* [59]	Molloplast B/heat-cured silicone	2	44
	Flexor/heat-cured silicone		39
Mese, A.* et al.* [60]	Mollosil Plus, autopolymerized silicone	12	29
	Molloplast B/heat-cured silicone		42
Polyzois, G.L.* et al.* [61]	EverSoft/heat-cured acrylic	10	27
	Super-Soft/heat-cured acrylic		78
Parr, G.R.* et al.* [62]	Luci-Soft/heat-cured silicone	10	42
	Tokuyama Soft/autopolymerized silicone		19
Kiat-Amnuay, S.* et al.* [9]	Novus/heat-cured polyphosphazene	11	33
	Luci-Soft/heat-cured silicone		38
	Molloplast B/heat-cured silicone		36
	Tokuyama Soft/autopolymerized silicone		22
	Permasoft/autopolymerized acrylic		18
Pavan, S.* et al.* [65]	Molloplast B/heat-cured silicone	6	35
	UfiGel P/autopolymerized silicone		19
	EverSoft, heat-cured acrylic		18
	Mucopren soft/autopolymerized silicone		27
Mancuso, D.N.* et al.* [67]	Dentusil/autopolymerized silicone	3	37
	UfiGel P/autopolymerized silicone		30
	UfiGel SC/autopolymerized silicone		32
Mante, F.K.* et al.* [14]	Permasoft/autopolymerized acrylic	4	29
	Tokuyama Soft/autopolymerized silicone		27
Chladek, G.* et al.* [52]	UfiGel SC/autopolymerized silicone	6	31
Kim, B-J.* et al.* [63]	Durabase/autopolymerized acrylic	6	29
	Dentusil/autopolymerized silicone		29
	GC Reline Soft/autopolymerized silicone		50
	GC Reline Ultrasoft/autopolymerized silicone		21
	Mucopren Soft/autopolymerized silicone		33
	Mucosoft/autopolymerized silicone		38
	Sofreliner Tough/autopolymerized silicone		35

Mante* et al.* [14] studied the changes in the hardness of sealed and unsealed LTSDL materials after immersion in different fluids for 90 days. Three different LTSDL materials (one silicone based and two acrylic based) were aged in artificial saliva, cleanser, and 50% ethanol. The hardness of the materials increased due to the use of the sealer alone. The SLTSDLs proved to be resistant to aging in all of the fluids, irrespective of sealer use. Although a marked increase in ALTSDL hardness was observed during aging in the cleansing solution, the hardness of the samples immersed in ethanol decreased dramatically by 70% after the first day until their complete degradation after seven days. The use of sealer was reflected by only slight changes in the hardness of the materials immersed in artificial saliva and cleanser. The hardness of the samples immersed in ethanol nevertheless changed considerably; however, this process was less dramatic than that for the unsealed materials because the hardness values decreased by approximately 15% and 70% after one and 30 days, respectively, and their complete degradation occurred after 80 days. The authors of this study postulated that the decrease in the hardness of the ALTSDLs immersed in ethanol was associated with the leaching of dibutyl phthalate from the material; this process might be responsible for the more rapid degradation of this type of lining in individuals who consume alcohol. The use of sealers can also reduce the surface degradation of soft liners subjected to mechanical brushing [68]. In conclusion, sealers appear to preserve the initial hardness of LTSDL materials; however, analysis of the biological resistance of sealed materials and of their resistance to cyclic mechanical loading constitutes an interesting direction for future research.

## 6. The Bond Strength to Denture Base Polymers

**Figure 3:**
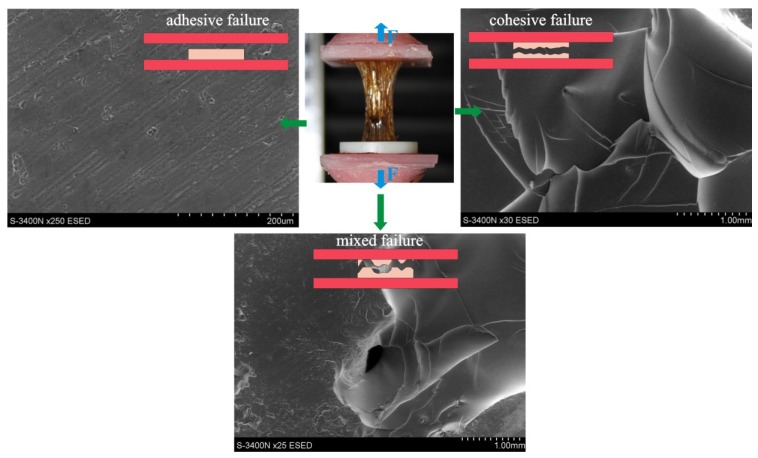
Various types of failures observed during bond strength tensile testing; the SEM images illustrate cohesive, adhesive and mixed (adhesive/cohesive) failures.

**Figure 4:**
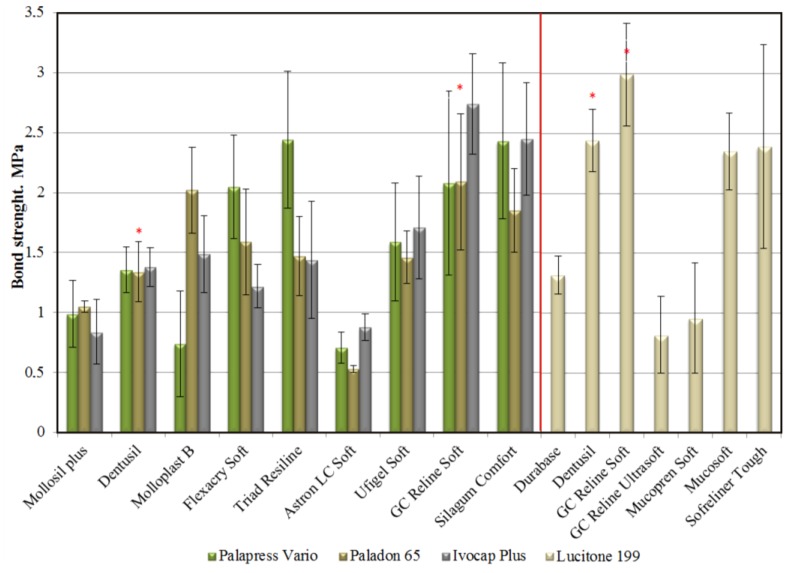
The tensile bond strength values obtained by Mutluay* et al.* [70] (left side), according to the ASTM standard, and by Kim* et al.* [63] (right side), according to the ISO standard. The diagram illustrates the differentiation of the results obtained using a similar methodology. The stars indicate the same LTSDL materials. The differences were the following: different denture base materials were used; in the study by Mautalay* et al.*, the denture base materials were wet ground using 1200-grit abrasive paper and were not stored, whereas Kim* et al.* used denture base materials that were wet ground using 500-grit abrasive paper and were stored at 37 ± 1 °C for 28 days.

An appropriate bonding quality to an acrylic denture base is a prerequisite for normal LTSDL functioning. Three established methods—peel testing, shear testing and tensile testing—are used to determine the bond strength of the lining to the acrylic base. However, the results of these laboratory tests have not fully reflected the actual bond strength of soft lining materials to the acrylic base under clinical conditions. The material is subjected to only one type of strain during each of these tests, in contrast to real-life conditions, under which it is exposed to the long-term effects of various levels of cyclic masticatory forces acting in the unstable environment of the oral cavity [69]. The peel test and shear test have rarely been used due to difficulties in interpreting their results [3,69,70,71,72]. Tensile bond strength tests, which are recommended by the ASTM [73] and ISO standards [55], are widely accepted. Both methods are very similar and require cross-linking of the samples inside a ring inserted between two plates composed of the denture base material (Figure 3), and these methods constitute a good starting point for scientific investigations. In some studies, different samples have been used, with the material placed between two cuboid-shaped blocks [54,74,75]. The different experimental protocols could affect the results of the bond strength tests; for example, thicker LTSDL layers exhibit lower bond strength values, and a faster cross-head speed results in higher tensile bond strength values and less elongation [63,69]. In addition, the type of denture base material, the preparation of its surface with different-grit abrasive papers or the aging of denture base material substrates before LTSDL application could affect the results [70]. The exemplary differences in the tensile bond strength results obtained using the ISO and ASTM methods are shown in Figure 4. A sample could also undergo cohesive or adhesive/cohesive failure types during tensile testing (Figure 3), and the strength of the lining material, rather than that of the bond, is determined under such conditions. Thus, the only finding of the test is that the bond strength exceeded the strength of the tested material, and this fact should be emphasized when drawing conclusions regarding the effects of environmental factors on the test outcomes. Therefore, the actual bond strength of LTSDLs to denture base polymers under clinical conditions would be better evaluated using an additional fatigue test that examines the effects of the simultaneous exposure of samples immersed in aging solutions to compressive and shear forces.

Mutluay* et al.* [70] demonstrated that the type of acrylic resin used exerted no effect on the mean initial tensile bond strength of silicone-based materials. This phenomenon resulted from the use of bonding agents that interacted with both the surface of the acrylic denture base and with the SLTSDL, thereby increasing the wetting of the substrate surface and impregnating its superficial layer with a mixture of polymeric components. Although the initial bond strengths of materials containing methacrylate groups were similar, their values differed depending on the denture base polymer, reflecting the effects of the resin and ALTSDL composition on the final strength. The authors emphasized the high prevalence of adhesion failures attributed to the inhomogeneity of the bonding area in the form of contamination and the internal stress resulting from volume contraction during the evaporation of the solvent and from the polymerization of the material components.

The tensile bond strength decreases with increasing aging time in various solutions, in association with the swelling and altered stiffness of the lining material and the formation of fissures and internal stress at the lining-denture interface; notably, the unfavorable effects of aging time are stronger for ALTSDL materials than for SLTSDLs and for cold-cured rather than heat-cured materials [60,70,75,76,77,78,79,80]. The effects of exposure to thermal cycles on the bond strength of LTSDLs depend on the chemical composition of the latter. Although the bond strengths of some materials decrease due to internal stress at a lining-denture junction, the strengths of others remain constant or even increase due to the continued cross-linking process [54,75,76,77,78,79,80,81].

The bond strength is also modulated by pretreatment of the denture base polymer surface. The rubbing of a monomer (MMA) into acrylic resin results in greater bond strength due to increased penetration of the chains of the acrylic resin into the ALTSDLs or into the bonding agent dedicated to SLTSDLs. In contrast, sandblasting results in decreased bond strength, most likely due to an increase in the internal stress on the lining-denture junction and the formation of indentations on its surface [82]. Furthermore, studies with radiolabeled solution revealed that, contrary to pretreatment with corundum abrasive, rubbing monomer into the denture base surface improves the tightness of the junction and prevents its penetration by liquid [83], which should be reflected by a greater bond strength. Lassila* et al.* [84] suggested that the strengthening of acrylic resin with glass fibers could decrease the bond strength resulting from the application of a bonding agent.

The peel test has been suggested to simulate the horizontal component of masticatory forces, but it generates a high risk of cohesive failure [69]. This test is considered to be more similar to real conditions than the tensile test because, in a clinical setting, debonding could be caused by stresses at the edges of the bonded soft lining, which can lead to the lifting and peeling of the material [71]. McCabe* et al.* [71] examined three SLTSDLs with different filler concentrations. The materials with lower concentrations exhibited greater compliance. The materials with the lowest compliance exhibited the lowest peel bond strength, while during tensile bond strength tests, this tendency was reversed. These researchers believed that the “lifting force” for a stiffer material was more likely to cause debonding because the stress was transferred to the interface area, while for a softer material, this force was more likely to deform the soft lining elastically and leave the interface area intact. A similar tendency for the same three SLTSDL materials in the peel test was observed by Tanimoto* et al.* [85]; however, after 30 additional days of water storage, lower peel resistance was recorded. Machado* et al.* [86] observed that the peel bond strength of two autopolymerized SLTSDLs was not affected by microwave disinfection. Sertgöz* et al.* [72] investigated the effects of thermocycling on the peel strength of six SLTSDLs; however, the results did not show any general tendencies as a function of the materials group: for one autopolymerized material, the peel strength decreased; for one autopolymerized material, the peel strength increased; and for the two autopolymerized and two heat-cured materials, the peel strength was unchanged.

The shear test is considered to be a useful method for testing bond strength because it is also more closely related to clinical settings than the tensile test. However, the stresses in soft linings are unevenly distributed and concentrated near the edges, and according to [69], the concentration increases with the specimen thickness, resulting in lower shear strength values. The cross-head speed can also affect the results [69]. Jagger* et al.* [87] reported that roughening of the surface produced only a nonsignificant increase in shear bond strength compared with a smooth surface. Al-Athel* et al.* [88] demonstrated that for the heat-cured SLTSDL Molloplas B, the shear bond strength during aging in distilled water decreased by more than 40% at a temperature of 37 °C after three months; at a temperature of 50 °C after one month, the tensile bond strength was changed by nearly 15%. Thus, the authors suggested that the results of long-term aging at 37 °C could be achieved in a shorter period at a higher temperature; however, this conclusion should be confirmed for other materials in further investigations. Hayakawa* et al.* [89] reported that the heat-cured ALTSDL Super-soft yielded the highest shear bond strength compared with two SLTSDLs and with autopolymerized ALTSDL. The authors indicated that the results were due to the high temperatures during polymerization and the similar chemical structures, which could facilitate the diffusion of methacrylate molecules from ALTSDL into the denture base material, thereby creating better bonding. In contrast, the autopolymerized ALTSDL exhibited rapid hardening, which did not allow the molecules to diffuse sufficiently into the denture base material; thus, the shear bond strength values were nearly halved. All of the tests were performed within 4 h after polymerization; thus, the authors did not consider the effects of aging in liquid, which could have been important when considering the high water sorption and solubility of Super-soft ALTSDL.

The other property related to forces acting on LTSDLs is the energy required to tear the material [5]. This property is dependent on the cross-head speed (tear rate), storage conditions and elastic properties of the LTSDL [5]. LTSDLs exhibit reduced tear strength after finishing and cleaning procedures where the risk of rupture is greater [90,91]. Yanikoğlu* et al.* [91] reported that the tear energies of three LTSDLs decreased with increased aging time, and the most destructive solution was a denture cleaner. No significant differences between the tear energy for microwave-activated and heat-activated Mollopast B silicone were observed by Baysan* et al.* [92]; however, a prolonged microwave irradiation time was observed to reduce the tear strength, perhaps due to the thermal shocks applied to the material. Landayan* et al.* [93] showed that 3000 thermal cycles with temperatures of 5 °C and 55 °C had no effects on the average tear force. Santawisuk* et al.* [10] reported gradual and significant increases in the tear and tensile strength after reinforcement with a hydrophobic silica filler.

## 7. Sorption and Solubility

Water absorption and leaching of LTSDL components occur simultaneously in the moist environment of the oral cavity. This phenomenon is reflected by changes in the mechanical properties, discoloration, swelling, odor, and easier bacterial adhesion to the lining’s surface [3,94]. Therefore, an ideal LTSDL material should neither absorb water nor contain soluble or leachable components [3]. The sorption, solubility and uptake of LTSDLs have been frequently studied; however, investigators have used varying experimental protocols, including different samples (thickness and diameter), aging times and aging solutions. The ISO standard requires samples with a thickness of 0.5 mm and diameter of 50 mm; however, thicker samples have often been used in previous studies, which could affect the results. In addition, different methods for sorption and solubility calculations have been used, such as percentage changes in mass [67,95] or mass changes per unit volume [55]. Additionally, in some studies, uptake during aging has been measured as a percentage weight change between the initial weight and the weight at a given time [96]. Thus, the results from different studies might be difficult to directly compare, but they illustrate the effects of various factors on the measured properties.

The water sorption of LTSDLs is dependent on the leachable components and on the hydrophilicity of the matrix. In ALTSDLs, the sorption of water is high even after a long period of time because of the leaching of plasticizers. This process creates locations for the formation of water droplets, and then, the droplets grow until their osmotic pressure reaches the same level as the external solution, or it is fixed by elastic forces [95]. Many factors, such as the degree of cross-linking, the polymer structure, and the presence of fillers or liquid, can affect the transport of liquid through elastomeric polymers [96]. The use of silane treatment fillers with coupling agents is a widely accepted method for altering the mechanical properties of elastomers; however, this method can also reduce water uptake. The lack of compatibility of the filler with the polymer can also cause the formation of voids at the interface and can lead to increased water absorption [96]. For instance, Waters* et al.* [97] analyzed the effects of incorporating three different surface-treated hydrophobic silica fillers into experimental RTV SLTSDL materials, and the authors observed a large reduction in sorption. They suggested that the reason for the high water sorption of the previous material could have been inappropriate surface treatment of the silica filler.

The interaction of LTSDLs with various liquids in long-duration tests was studied by Liao* et al.* [98]. Two SLTSDLs (heat cured and autopolymerized), one autopolymerized (ALTSDL EverSoft) and one temporary heat-cured soft lining (Vertex soft) were investigated in distilled water, artificial saliva, 3% aqueous acetic acid, 10% ethanol, 50% ethanol and coconut-oil-based liquids. Both silicones, despite the slightly different results, behaved similarly, and the authors concluded that the uptake of water occurred first through the polymer matrix, and next, the greater part of the uptake was caused by the presence of water-soluble moieties in the filler. The uptake changes obtained in the coconut-oil-based liquids suggested that some soluble component was extracted. Both soft acrylics behaved similarly in the 50% ethanol solution. First, a rapid increase in uptake was observed, which was explained by the absorption of ethanol. Next, the mass change was reversed, most likely due to the domination of leaching of the plasticizer, which is soluble in ethanol. Finally, the absorption dominated again. The processes of absorption and extraction occurred simultaneously, with detrimental effects on materials based on poly(ethyl methacrylate).

When exposed to thermal cycles, ALTSDLs exhibit greater increases in sorption and solubility than SLTSDLs [63]. Cyclic mechanical loading has no effects on the sorption and solubility of SLTSDLs [99].

The sorption and solubility of ALTSDL materials are markedly higher than those of SLTSDLs [3]. Yanikoglu* et al.* [100] observed a medium-independent increase in the sorption and solubility of SLTSDLs over 16 weeks of aging. Although the sorption and solubility of ALTSDLs were not subjects of this study, these parameters of acrylic tissue conditioners varied markedly depending on the aging solution. Parker* et al.* [7] analyzed polyphosphazene-based LTSDLs and observed that the sorption of these soft lining materials in distilled water was higher than in artificial saliva; this phenomenon was due to osmotic processes. Based on this finding, these authors postulated that sorption and solubility should not be determined in distilled water but in ionized solutions because the results achieved under such experimental conditions would more closely resemble those observed in real conditions. Although this approach is appropriate, standardization of the chemical composition of the aging solution would be required to allow for comparisons between the results observed in various studies. Brożek* et al.* [13,14,15,16,17] reported that cleaning agents caused the leaching of fewer compounds than artificial saliva, which could indicate that the cleansers caused fewer chemical changes in LTSDLs than the natural environment.

## 8. Viscoelastic Properties

Soft denture liners are subjected both to instantaneous pressure during mastication and to continuous pressure, of a smaller magnitude, of the oral soft tissue during resting [101]. In several studies [102,103,104,105], it has been stated that an ideal soft lining material should have the same viscoelasticity as the alveolar mucosa. However, soft lining compensates for deficiencies of the mucosa, and mimicking the mechanical behavior of their viscous flow causes a reduction in the rate of shape recovery, which is considered to be unfavorable during transfer of the cyclic mastication load [96,106,107,108]. In general, time-dependent deformation can be regulated with the glass transition temperature by adding plasticizers to reduce intimate contact between the polymer chains [109].

The viscoelastic properties of LTSDLs can be determined based on creep curves obtained during the loading and unloading of the samples with a flat-ended cylindrical-shaped penetrator. The immediate and delayed compliance of the tested material and its ability for elastic recovery were determined [105,110,111]. McCabe* et al.* [112] applied a load of 100 g for 30 s using a 1-mm-diameter penetrator and then unloaded the samples to 4.31 g for the next two minutes for recovery. Although a significant reduction in the compliance values was observed after immersing the ALTSDL materials in distilled water, the aging of SLTSDLs did not modulate their elasticity [111,112]. The elastic recovery of SLTSDLs and ALTSDLs required 1–2 s and more than 30 s, respectively [112]. Thus, ALTSDLs could exhibit limited elastic recovery during chewing, reflected by a temporal decrease in their ability to transmit the load to the mucosal membrane. In their study using a creep-meter, Muraoka* et al.* [99] showed that cyclic loading had no effects on the viscoelastic properties of SPDLs. The satisfactory viscoelastic properties of SLTSDLs exposed to cyclic loading were also confirmed by Tamura* et al.* [102].

Only a few authors have analyzed the viscoelastic properties of soft lining materials using the dynamic mechanical analysis (DMA) technique. During testing using this method, the samples are exposed to cyclic loading to determine the values of the storage modulus *E*’, which is an equivalent of the Young’s modulus of an elastic material; the loss modulus *E*’’, which is associated with the dissipative (viscous) component; and the damping factor, also called the loss tangent (tan δ),* i.e.*, the *E*’’/*E*’ ratio (the amount of energy dissipated during the cycle) [4]. A higher complex modulus E* (MPa) implies that the material is difficult to deform under the investigated frequency:

E* = [(*E*’)^2^ + (*E*’’)^2^]^1/2^ (1)

According to this formula, for a higher loss modulus, the storage modulus must be lowered to maintain good softness and rapid elastic recovery (unchanged E*).

Initially, the ALTSDLs at a frequency of 1 Hz and under the load caused by 0.5% of cyclic strain [4] are generally characterized by high values of the damping factor, and these parameters undergo evident unfavorable changes during aging in distilled water. In contrast, the SLTSDL materials are characterized by more stable properties during aging and lower values of the damping factor and *E*’ [4,103,108]. According to Santawisuk* et al.* [103], low damping factor values of commercially available SLTSDL materials are not preferred because LTSDLs with higher damping factors possess a greater ability to absorb functional stress. These authors used a simple laboratory instrument to compound from 2% to 10% of the hydrophobic surface-treated silica filler into experimental RTV silicone, which increased both the loss modulus and damping factor [103]. The loss modulus *E*’’, measured at a relatively small strain value of 0.27% and frequency of 1 Hz, increased from an initial value of 0.061 MPa to 0.206 MPa with increasing filler content. Although the efficiency of LTSDLs to dissipate energy increased with increasing filler content, the storage modulus *E*’ also increased from 1.03 MPa to 2.21 MPa. The storage modulus, despite the increase, remained lower compared with two other investigated commercial silicones. Abe* et al.* [113] reported that the storage modulus, investigated using the non-resonance forced vibration method (1 Hz, 37 °C) after 1 day of wet storage (37 °C), also increased with the silica filler concentration in a SLTSDL. In addition, the complex modulus increased from 1 MPa to 3.5 MPa upon increasing the filler concentration from 18% to 37% for GC Reline materials and from 1 MPa to 1.5 MPa for Sofreliner upon increasing the filler concentration from 6% to 20%. A complex modulus of 3 MPa was reported for Mucopren with 30% filler, whereas the loss modulus decreased with increasing filler content. Based on these contrasting results [103,113], it can be hypothesized that the obtained effect on the loss modulus was dependent on the efficiency of compounding the filler and on the chemical bonding between the surface-treated filler and the polymer matrix. In [96], a reduction in tan δ was observed with increasing concentrations of silanized silica in experimental elastomer/methacrylate soft linings based on blends of isoprene–styrene block co-polymer and mixtures of methyl methacrylate and 1,6-hexanediol dimethacrylate. The addition of silica increased the storage modulus values, which remained at satisfactorily low levels only for materials with dimethacrylate monomer concentrations ranging from 10% to 20%.

It has been widely acknowledged that soft denture liners are used to cushion (damping) transmitted forces and relieve pain [101,103,104,105,106,107,108,109,110,111,112,113]. The cushioning of a load is not the same as relieving the load [5], which appears to be the source of confusion. If a denture fits and sits firmly on the foundation, the average pain pressure threshold of 630 kPa [114,115] is not reached even if the thin and hard mucous membrane bears heavy mastication loads [116]. Under a lateral mastication load, the same denture does not sit firmly on the foundation and moves and tilts despite the balancing contact working well [117,118]. Beneath the tilted denture, the area of support is small, and the pressure is several-fold greater than the average pain threshold [116]. With an unfavorable foundation, the pressures beneath the denture exceed the pain threshold even after implantological stabilization [119,120], and the soft lining is still required. Quantitative data showing the mechanism of pain creation associated with masticatory load transfer with hard dentures [116] indicate that the increase in the load-bearing area beneath the inclined denture should reduce pain. There has been agreement with suggestions that the elastic deformation of the soft lining under the dynamic behavior is the key feature [106,107,108,121] but not only because of more even pressure. The softer denture lining, in terms of *E*’ at 1 Hz, may experience greater elastic strain, resulting in an increase of the contact surface beneath the denture. Viscous flow of the soft lining only results in a further 20% decrease of the stress in the soft tissue [122,123]. The relieving effect differs from load absorption, which increases with greater damping, in terms of higher *E*’’, indirectly converting mechanical work into heat in the bulk of the material but worsening elastic recovery during the cyclic mastication. However, there are opinions [113] that softness can reduce denture stability and lead to pain from malocclusion, although this result could equally be due to the increase in shear stress at the slopes of alveolar processes [124]. Under the vertical occlusal load of a well-fitted denture to the foundation, after the introduction of a soft lining, more of the load is distributed from the crest to the slopes, and the tissues at the sides undergo greater distortional deformation despite the pressure being lower. The results of numerical analyses should be interpreted carefully because the commonly used criterion of maximal distortional strain energy (Huber-Mises stress) is a determinant of shear stress. Investigations of load transfer in soft tissue [125,126,127] have indicated that shear stress and pressures should not be analyzed as sole criteria because such an analysis can lead to misinterpretation. This finding was also observed in mucous membrane denture foundations [124], where the shear stresses can increase two-fold after the use of a soft relining. Nevertheless, the capacity of the soft lining for stress relaxation by viscous flow is helpful under continuous pressure during resting. The denture manufacturing inaccuracies appear to have been the primary cause of misfit and patient complaints [22,128,129], although Murata* et al.* [101] reported that foundation changes could cause misfit and continuous pressure. At the same time, Cha* et al.* [130] observed that the yielding properties of ALTSDLs affected the dimensions. Whether the viscous flow of a soft material can compensate for the inaccuracies of a denture to a greater extent than introducing a new material, which may introduce new inaccuracies, should be considered. It is necessary to consider whether pure elastic strain is sufficient to compensate for the inaccuracies, particularly when viscous flow might cause permanent deformation during function and loss of fit. In conclusion, due to the nonlinear mechanical behavior of soft linings, dynamic tests corresponding to chewing must be performed at a frequency of approximately 1 Hz and at a stress on the level of the pressure pain threshold of the mucous membrane,* i.e.*, greater than 630 kPa [114,115]. In contrast, creep tests should be performed under pressures of 50–275 kPa, which can cause ischemia and ulcers in soft tissues [131,132,133,134].

The compositions of materials must be assessed with regard to aging, which has considerable effects on viscoelasticity due to the complex interaction between water uptake and oxidation [109]. Water present in a lining can act as a “filler” by stressing the surrounding polymer matrix and, as a result, stiffening the material [109], with the largest changes in the viscoelastic properties of silicones after 60 days of wet storage [113].

## 9. Color Stability

Color stability is normally examined during enhanced aging [135]. Nicotine exerts a particularly detrimental effect on the color of LTSDLs, causing their darkening [136], as confirmed by the previously mentioned results of clinical studies [7,8,9,10,11,12,13,14,15,16,17,18,19,20,21]. Tea, coffee, and wine cause less pronounced discoloration of LTSDLs. Notably, silicone-based materials are characterized by more stable color than acrylic materials in various aging solutions such as coffee or tea [137,138]; similarly, the color of heat-polymerized materials is more stable than the color of autopolymerized materials [138,139,140]. Similar relationships were documented during the aging of LTSDL materials in food-colorant solutions; however, only a temporary acrylic liner was compared with an SLTSDL [59]. Denture cleansers can remove the discoloration of LTSDLs caused by food-colorant solutions; however, staining can still persist at a visible level after cleaning, and the cleansers vary in terms of their effectiveness [141]. Cleaning agents did not cause significant changes in the elasticity modulus of SLTSDL materials during 28 days of aging [17]. The color of LTSDLs can also be modulated by the temperature of the water in which a denture cleaner is dissolved [142] or by the use of thermal cycles [143].

## 10. Summary and Conclusions

Due to their favorable mechanical and performance properties, LTSDL materials guarantee a significant increase in the comfort of denture wearers. However, the durability of these materials under clinical conditions is limited, primarily due to their low resistance to colonization by *C. albicans*. The preliminary results of several studies have suggested that the microbiological properties of LTSDLs can be improved through the use of fillers with antimicrobial properties, such as nanosilver; however, the latter causes discoloration of the material. Therefore, further research on the potential applications of other antimicrobial fillers, such as white-colored ceramics, is needed. In future studies, special emphasis should be placed on the stability of the potential antimicrobial effects, which should last for at least a few months.

Considering the migration of liquids and yeast inside LTSDL materials, particularly to the bonding area, an improvement in microbial resistance will supposedly also reduce another important problem, namely, the debonding of SLTSDLs from the denture base.

Studies have demonstrated that, in contrast to ALTSDLs, the hardness of SLTSDL materials is relatively stable over time. In addition, the sorption and solubility of SLTSDLs are significantly lower than those of ALTSDLs.

Moreover, unlike ALTSDLs, the viscoelastic properties of SLTSDL materials are appropriate and stable over time. The color of LTSDLs is unstable over time and can be altered by many factors, particularly by nicotine.
