# Correction: Long-Term Soft Denture Lining Materials. Materials 2014, 7(8), 5816-5842

**DOI:** 10.3390/ma8063791

**Published:** 2015-06-23

**Authors:** Grzegorz Chladek, Jarosław Żmudzki, Jacek Kasperski

**Affiliations:** 1 Division of Materials Processing Technology, Institute of Engineering Materials and Biomaterials, Silesian University of Technology, ul. Konarskiego 18a, Gliwice 44-100, Poland; E-Mail: jaroslaw.zmudzki@polsl.pl; 2 Department of Prosthetic Dentistry, Medical University of Silesia, pl. Akademicki 17, Bytom 41-902, Poland; E-Mail: kroczek91@interia.pl

In the published manuscript “Long-Term Soft Denture Lining Materials. Materials 2014, 7(8), 5816-5842” [1] we detected that in three places reference numbers were inserted incorrectly due to an error in the editing. We apologize for any inconvenience this may have caused.

Page 5819, Section “3. Clinical Effects of Long-Term Soft Denture Linings”

It is: Although the longevity of SLTSDLs was confirmed under clinical conditions, the presence of discoloration and odor was frequently reported among tobacco smokers [7,8,9,10,11,12,13,14,15,16,17,18,19,20,21,31].

Should be: Although the longevity of SLTSDLs was confirmed under clinical conditions, the presence of discoloration and odor was frequently reported among tobacco smokers [20,21,31].

Page 5820, Section “3. Clinical Effects of Long-Term Soft Denture Linings”

It is: Thus, LTSDLs constitute a prospective group of dental materials, and previously reported clinical problems with LTSDLs [7,8,9,10,11,12,13,14,15,16,17,18,19,20,21,31,32] justify further research aimed at improving their properties.

Should be: Thus, LTSDLs constitute a prospective group of dental materials, and previously reported clinical problems with LTSDLs [20,21,31,32] justify further research aimed at improving their properties.

Page 5833, Section “9. Color Stability”

It is: Nicotine exerts a particularly detrimental effect on the color of LTSDLs, causing their darkening [136], as confirmed by the previously mentioned results of clinical studies [7–21].

Should be: Nicotine exerts a particularly detrimental effect on the color of LTSDLs, causing their darkening [136], as confirmed by the previously mentioned results of clinical studies [20,21].
